# Effects on health and antioxidant status of oral supplementation of hazelnut skin extract in dairy calves with neonatal diarrhea: a randomized clinical trial in hospitalized patients

**DOI:** 10.1080/01652176.2026.2691428

**Published:** 2026-10-14

**Authors:** Antonio Boccardo, Martina Sannazzaro, Giulia Sala, Elisa Gazzola, Ilaria Martucci, Giulio Curone, Marta Castrica, Vincenzo Ferrulli, Claudio Forte, Rosangela Odore, Giulia Gardini, Davide Pravettoni

**Affiliations:** a Department of Veterinary Medicine and Animal Sciences, University of Milan, Lodi, Italy; b Department of Veterinary Sciences, University of Turin, Grugliasco, Italy; c Department of Comparative Biomedicine and Food Science, University of Padua, Legnaro, Italy

**Keywords:** Hazelnut, polyphenols, calves, neonatal diarrhea, therapy, green economy, by-products, bovine medicine

## Abstract

Neonatal calf diarrhea (NCD) is a major contributor to morbidity and mortality in calves. Growing concern about antimicrobial resistance has spurred interest in phytotherapeutic alternatives, particularly polyphenol-rich extracts. Hazelnut skin (HS), a by-product of the confectionery sector, is a sustainable and underutilized source of polyphenols; however, its therapeutic efficacy in calves with NCD remains insufficiently investigated. This randomized, parallel-group clinical trial evaluated the effects of oral HS extract supplementation as an adjunct to standard medical therapy in hospitalized dairy calves with NCD. Sixty female Holstein calves were randomly allocated to a control group (CTRL; *n* = 30) or a treatment group (HS; *n* = 30) receiving 10 g/day of HS extract with milk for 5 days. All calves received standardized clinical management, including fluid therapy and antimicrobial administration. Clinical parameters, milk intake, body weight, hospitalization outcomes, sepsis scores, and blood oxidative stress biomarkers were monitored. No significant differences were detected between groups for clinical recovery, fecal score, hydration status, milk intake, weight gain, hospitalization duration, mortality, or oxidative stress markers. Clinical improvement over time was evident in both groups and was attributable to standard therapy. In conclusion, short-term oral supplementation with HS extract did not confer additional clinical or antioxidant benefits when added to conventional treatment in calves with NCD.

## Introduction

Neonatal calf diarrhea (NCD) is a prevalent condition that affects newborn calves and poses significant challenges to the dairy industry due to its associated mortality rates and treatment costs (Windeyer 2014; Urie et al. 2018; Roblin et al. 2023). This disease can result in dehydration, metabolic and electrolyte imbalances, potentially escalating to emergencies that may include neurological, septicemic, and cardiac complications (Trefz et al. 2017, 2018; Pas et al. 2023). Effective treatment strategies focus on restoring balance by administering intravenous and oral electrolyte solutions, tailored to the clinical severity of the condition (Constable et al. 2021), thus improving recovery outcomes and reducing long-term effects in surviving calves (Trefz et al. 2012; Boccardo et al. 2019). In contrast, the use of antimicrobials in the treatment of NCD has raised concerns and prompted reevaluation of their role in this disease (Gomez et al. 2017; Eibl et al. 2021; Gomez et al. 2021). Given the global reevaluation of antimicrobial use, particularly in farm animals, there has been growing interest in unconventional methods to improve the health status of food animals in recent years. One notable approach is phytotherapy, which utilizes plants or extracts known for their pharmacological properties or beneficial chemical constituents, such as polyphenols (Serra et al. 2021). Polyphenols are recognized for their significant antioxidant and anti-inflammatory properties, which help manage oxidative stress and stimulate endogenous protective enzymes (Cuevas et al. 2013). Furthermore, polyphenols modulate immune responses and intestinal microbiota while exhibiting antibiotic, antifungal, antiviral, and anti-helminthic properties (Lavecchia et al. 2013; Turini et al. 2022; Nabi et al. 2023). The increasing popularity of phytotherapy can be attributed to the ‘natural’ and ‘ethical’ origins of these compounds. A considerable number of phytotherapeutic agents are effectively sourced from the waste generated by agro-industrial food processing that could be reintegrated into zootechnical production cycles. Hazelnuts (*Corylus avellana*) present intriguing opportunities in this context. This product primarily serves the confectionery industry, which generates a noteworthy by-product: hazelnut skin (HS) that can be processed to yield an extract rich in phenolic compounds known for their antioxidant and antimicrobial properties (Capaldi et al. 2025).

Numerous studies have investigated the effects of both in vivo and in vitro phytotherapeutic solutions to evaluate their potential health benefits for food animals (Ayrle et al. 2016; Olagaray and Bradford 2019). These studies often highlight the preventive effects of phytotherapeutic compounds; however, there is a notable lack of clinical studies evaluating the efficacy of phytotherapeutics as an ancillary treatment for diseases in cattle. This lack of research may stem from the need for high concentrations of extracts and essences to achieve curative effects (Smulski et al. 2020). Notably, the impact of phytotherapeutic supplementation on diarrheic dairy calves has only been studied in two trials. Chestnut tannins were found to reduce the duration of diarrhea in calves by four days (Bonelli et al. 2018). Similarly, multi-essence feed additives were shown to improve weight gain in treated calves compared with those receiving antimicrobials, with a concomitant reduction in recovery time from diarrhea in the treated group (Wang et al. 2022).

To date, no research has examined the effects of HS extract supplementation in diarrheic dairy calves, particularly its potential benefits for health and antioxidant status. To fill this gap, we conducted a randomized clinical trial that involved the longitudinal monitoring of diarrheic calves admitted to a veterinary teaching hospital. We employed standardized clinical scoring to evaluate differences in health improvements between calves receiving oral HS extract and those that did not during hospitalization. Furthermore, we assessed levels of derivatives of reactive oxygen metabolites (d-ROMs) and serum antioxidant capacity using the oxygen-adsorbent test (OXY) in both the treated and control groups. We hypothesized that administering HS extract would improve health status, facilitate recovery, and strengthen antioxidant defenses.

## Material and methods

### Study design, ethics approval

This consecutive, parallel, randomized clinical trial with longitudinal follow-up of the enrolled calves was approved by the institutional animal welfare organization of the University of Milan (approval number 93_2023) and conducted following CONSORT guidelines at the Clinic for Ruminant and Swine of the Veterinary Teaching Hospital of the Department of Veterinary Medicine and Animal Sciences (DIVAS), University of Milan, from March 2023 to June 2024.

### Study population, disease management, and setting

This study focused on a population of diarrheic calves that were admitted to the Clinic of Ruminants and Swine, part of the Department of Veterinary Medicine and Animal Sciences at the University of Milan (45°18′16″ N 9°28′20″ E). Upon admission, each patient underwent a comprehensive clinical examination, including a systematic thoracic ultrasound assessment performed based on the ventral landmarks described in previous studies (Ollivett et al. 2015) using a portable unit (Draminski Blue, Draminski Ultrasound Scanners, Sząbruk, Poland), a blood sample collected in a 9-mL tube without anticoagulant from the jugular vein used to determine serum total protein (STP) concentration with a handheld refractometer (model MR514ATC, Milwaukee srl, Gallarate, Italy), and a further blood sample for venous blood-gas analysis (EPOC Vet, Epocal Inc, Ottawa, Canada) anaerobically collected from the jugular vein into a disposable heparinized 2.5 mL syringe. Additionally, each calf was weighed using an electronic scale (Omega Twin 3, Omega Bilance S.r.l., Varese, Italy). Thoracic ultrasonography was selected as the reference diagnostic tool for bronchopneumonia exclusion, as it represents one of the most accurate methods for detecting lung consolidation in pre-weaned dairy calves (Berman et al. 2019).

Detailed information was recorded in the medical records, including a thorough history of the onset and duration of clinical signs observed before admission.

We evaluated calves aged up to 28 days that were admitted to the clinic with a prior diagnosis of NCD, as well as those presenting with clinical manifestations suggestive of NCD – such as altered mentation, a documented history of anorexia, impaired ability to maintain a quadrupedal stance, ataxia, or a reduced suckling reflex – were included in the evaluation. Inclusion criteria mandated that all calves exhibit clinical evidence of NCD, specifically watery feces, as defined by a fecal score of ≥2 (Renaud et al. 2020).

Following admission, calves were managed according to a standardized hospitalization and treatment protocol based on clinical categorization (Table 1) and blood gas results (Boccardo et al. 2017). Briefly, calves exhibiting a strong suckle reflex, a demeanor score of ≥4, a dehydration score of ≤1, and a base excess of up to −8 mmol/L were administered 1 L of oral rehydration solution (ORS). This solution contained 4 g of sodium chloride, 20 g of dextrose, 3 g of potassium bicarbonate, and 3 g of sodium propionate. The ORS was administered three times within the first 24 h, between milk feedings. Calves with a demeanor score of <4, dehydration >1, or a base excess of <−8 mmol/L received a constant drip infusion of 5 L of isotonic saline enhanced with 8.4% sodium bicarbonate (NaHCO_3_), calculated according to Lorenz and Vogt (2006), at a rate of 40 mL/kg/h. For calves exhibiting severe dehydration (≥score 2), an additional 5 L bag of isotonic saline was administered at a slow infusion rate of 10 mL/kg/h. Calves that had not completed the ORS at admission or between milk feedings were given a constant-rate infusion of 5 L of isotonic saline spiked with 8.4% NaHCO_3_(based on their base excess at admission) at a rate of 10 mL/kg/h. If any calves showed deterioration or failed to improve in hydration, demeanor, or suckle reflex scores within 24 h after treatment – or if they deteriorated after an initial improvement – they underwent an additional venous blood gas analysis. The non-responsive calves received additional intravenous fluid therapy tailored to their current acid-base balance and dehydration status. Furthermore, calves with a history of malnutrition or those not fed milk for more than 12 h were administered 400 mL of a 50% glucose solution combined with saline solution. All calves received an intravenous dose of 2.2 mg/kg flunixin meglumine (Alivios®, Fatro S.p.A., Ozzano dell'Emilia, Italy), along with 0.06 mg/kg of vitamin E and selenium (Selevit®, Fatro S.p.A., Ozzano dell'Emilia, Italy) and 10 mg/kg of thiamine (Dodicile®, Fatro S.p.A., Ozzano dell'Emilia, Italy) given parenterally.

**Table 1.** The clinical score used to evaluate the demeanor, degree of dehydration, strength of the suckle reflex and fecal score in diarrheic calves admitted to the Clinic of Ruminants and Swine, part of the Teaching Hospital within the Department of Veterinary Medicine and Animal Sciences (DIVAS) at the University of Milan.

Score	Clinical signs
**Demeanor**	
5	Standing securely without assistance, curious, alert
4	Standing up after encouragement, weak, ‘sad calf’
3	Sternal recumbency, standing after lifting, ‘drunken gait’, insecure posture
2	Permanent sternal/costal recumbency
1	Lateral recumbency, comatose
**Dehydration**	
0	Normal hydration, upper eyelid skin tent
1	Moderate dehydration, eyeball slightly sunken (1–2 mm), or upper eyelid skin tent > 2 s
2	Obvious dehydration, sunken eyes (3–4 mm), upper eyelid skin tent > 5 s (estimated loss of body mass 6%–8%)
3	Severe sunken eyes with an easily perceptible distance between the eyeball and the eyelid (≥5 mm), cold legs and oral cavity, dry mouth and nose, upper eyelid skin tent persists (estimated loss of body mass ≥ 9%)
**Suckle reflex**	
0	Strong
1	Weak
2	Absent or chewing movements
**Fecal score**	
0	Normal (firm but not hard)
1	Soft (does not hold form, piles but spreads slightly)
2	Runny (spreads readily)
3	Watery (liquid consistency, splatters)

Amoxicillin was selected as the empirical first-line antimicrobial treatment according to the routine clinical practice of the Ruminant and Swine Clinic and based on previous literature supporting its use in calves with diarrhea and systemic clinical signs (Berchtold 2009).

During their hospitalization, calves were housed in individual mobile hutches (OpenTop, CalfOtel) within a dedicated indoor room comprising 10 spaces, maintained at a controlled temperature of 18 °C/22 °C, specifically for calves with NCD. Freshwater, hay, and pellet calf starter were provided *ad libitum*. Calves were monitored twice daily through comprehensive clinical examinations and were given 2 liters of milk replacer (Solvor MG + Instant®; Cargill) three times daily (8 a.m., 2 p.m., and 6 p.m.), initiated after fluid therapy or whenever their clinical condition allowed (indicated by adequate demeanor and an improved suckle reflex score). The refused milk was measured and recorded.

Calves that survived were discharged from the Clinic in a healthy state, characterized by normal hydration and demeanor scores, a good appetite, and normal fecal consistency for at least two consecutive days.

### Sample size calculation

The number of diarrheic calves used (60, 30 per group) was the minimum required to detect a statistically significant treatment effect. This calculation was based on the NCD prevalence and mortality reported in the literature. The prevalence of NCD in the United States and Europe was approximately 19%, with a mortality rate of 20% (National Animal Health Monitoring System (US) 2010; Meganck et al. 2015). A two-sample *t*-test was conducted using G*Power (Version 3.1; Heinrich-Heine-Universität, Düsseldorf, Germany) to determine the required sample size. For this calculation, a small effect size (0.3), a type I error rate (α) of 5%, a 95% confidence interval, and a test power of 80% were applied. The resulting sample size was 60 animals.

### Eligibility criteria

This study examined hospitalized calves suffering from NCD, which met the following criteria:

– Only female Holstein heifers were included.
– Upon admission, calves were required to present with a fecal score of ≥2 (Renaud et al. 2020).

To minimize potential biases in the results, several concomitant diseases were established as exclusion criteria at the time of admission. However, these conditions were reevaluated if they manifested during the study or hospitalization period. The diseases considered for exclusion included:

– Arthritis (calves exhibiting swelling in one or more joints were excluded).
– Meningitis (calves that displayed tonic-clonic seizures, exaggerated cranial nerve reflexes, or opisthotonus were excluded).
– Umbilical disorders (calves were excluded if there was swelling of any internal structures – arteries, urachus, or vein – or the presence of neoformations immediately dorsal to the inner umbilical ring, with or without stump abnormalities).
– Pneumonia (diagnosed via thoracic ultrasonography, with lesions of 1 cm² or greater serving as the diagnostic threshold).
– Cachexia (clinically diagnosed in cases of severe reduced body condition with sarcopenia assessed at the level of the spine of the shoulder).

Only calves that adhered to the outlined treatment protocol were considered eligible for participation in the study. Furthermore, most calves admitted to our facility present with severe clinical signs, so we decided to include only calves requiring intravenous infusion therapy to form two homogeneous groups (only calves with at least one of the following criteria were enrolled: demeanor score <4, dehydration >1, or base excess <−8 mmol/L). However, calves that had already received intravenous infusion therapy on the farm were excluded from the study.

### Randomization method and grouping

After determining a minimum required sample size of 60 calves, we created an a priori randomization list using an Android application (Randomizer, Darshan Institute of Engineering and Technology, Rajkot, India). This list enabled us to consecutively assign each eligible diarrheic calf admitted to either a control group (CTRL, *n* = 30) or a treatment group (HS, *n* = 30).

All calves were subjected to the same standardized hospitalization and treatment protocol utilized in the clinic. The sole distinction between the two groups was that the HS group received 10 g/d of HS extract supplementation, mixed into the 8 a.m. milk feeding, for 5 days, beginning with the first meal offered after their admission. If any portion of this feeding was not wholly consumed, replacement milk containing HS was stored in the refrigerator and offered again during other meals throughout the day. This dose and protocol were selected based on previous studies using polyphenol-rich plant extracts in calves and other livestock species, while also considering feasibility under clinical and potential field conditions during the acute phase of disease (Bonelli et al. 2018; Wang et al. 2019; Turini et al. 2022).

### Hazelnut skin extract characteristics

The hazelnut skin extract administered to the HS group was obtained using green extraction methods (GPES) as outlined by Capaldi et al. (2025). Specifically, this study utilized the batch 4 extract (B4E) from a batch supplied by a feed company that sources hazelnut skins from various suppliers (Capaldi et al. 2025). The extraction process employed was microwave-assisted subcritical water extraction, yielding nearly 25%. Quantitative analysis of the HS extract indicated a total phenolic content of 778.4 ± 16.0 mg gallic acid equivalent (GAE) per gram of dry extract. The composition of phenolic compounds consisted of 1.36 ± 0.031 mg/g of phenolic acids, 14.84 ± 0.06 mg/g of flavan-3-ols, and 0.51 ± 0.003 mg/g of flavonols. As a result, the HS group in this study received a total daily polyphenol dose of 7784 mg per calf. Furthermore, antioxidant activity, assessed using the 1,1-diphenyl-2-picrylhydrazyl method, yielded a value of 4.51 mmol TE eq./g of extract, while the oxygen radical absorbance capacity test indicated a value of 6.86 mmol TE/g for B4E.

### Study protocol and clinical status evaluations

Each calf underwent a clinical assessment from admission through the sixth day of hospitalization (T0, the day of hospitalization; T6, six days after hospitalization), comprising seven evaluations. In addition to the clinical signs detailed in Table 1, all calves underwent daily (at admission to T6) sepsis probability (%) evaluations using the tables and the categorizations from Fecteau et al. (1997) (Supplementary Table 1).

Clinical evaluations were systematically performed each morning immediately following the initial feeding. All clinical assessments were conducted by one of the principal authors affiliated with the Clinic for Ruminants and Swine (AB, DP, EG, IM). Throughout the duration of the study, the first author (AB) systematically monitored the clinical scores to ensure both accuracy and consistency. Furthermore, each calf was weighed daily from the time of admission until day six, prior to the morning meal. Upon admission, fecal samples were collected per rectum from both treated and control calves, placed in sterile containers, and promptly transported to a laboratory for bacteriological culture, including evaluation for *Cryptosporidium* spp., and PCR testing for Rotavirus and Coronavirus spp.

### Blood oxidative stress assay

Each enrolled calf from both groups underwent venous blood sampling via jugular venipuncture with a 20-gauge hypodermic needle at enrolment and the end of HS (or control) administration (day 6 after hospitalization). The collected blood was stored in a sterile Vacutainer tube without an anticoagulant. Samples were centrifuged at 20 °C for 10 min at 900 g within 4 h of collection. The obtained serum was transferred into 2 mL Eppendorf tubes and stored at −80 °C until the blood antioxidant status of all calves was evaluated at the study's conclusion.

Serum antioxidant capacity was assessed using the OXY-Adsorbent test (Diacron International, Grosseto, Italy). Briefly, serum samples were diluted 1:100 in distilled water and mixed with a hypochlorous acid (HClO) solution. After incubation at 37 °C for 10 min, a chromogenic solution was added, and absorbance was measured at 505 nm. Results were expressed as µmol of HClO per mL of sample (Pirro et al. 2015). The determination of d-ROMs (d-ROMs Test, Diacron International Srl, Grosseto, Italy) was performed according to the manufacturer's instructions and as described by Sauerwein et al. (2020). Briefly, a mixture of acetate buffer and chromogenic solution was added to each sample, and sera were incubated at 37 °C for 30 min. Absorbance was measured at 505 nm, and the results were expressed in UCarr (1 UCarr corresponds to 0.08 mg H_2_O_2_/dL), using a human standard serum as a calibrator after subtracting the blank.

### Statistical analysis

All statistical analyses were performed using IBM SPSS Statistics (version 29). Continuous variables were evaluated for normality using the Shapiro–Wilk test. Single variables with normal distributions were summarized as mean ± standard deviation (SD) and compared between groups using independent-samples *t*-tests. Non-normally distributed single variables were summarized as median and interquartile range (P25–P75) and compared using the Mann–Whitney U test. Categorical variables were expressed as frequencies and percentages and compared between groups using the chi-square test. Descriptive statistics for repeated measures were generated separately for each group at each time point (T0–T6), depending on the nature of the variables and their distributions.

Because clinical outcomes were heterogeneous in scale and distribution, distinct Generalized Estimating Equation (GEE) models were constructed for each variable. Calf ID was specified as the subject variable to account for repeated measurements, and time was included as a within-subject factor. Continuous outcomes (body weight, milk intake, rectal temperature, OXY, d-ROMs) were analyzed using GEE with a log link and gamma distribution. Ordinal clinical outcomes – ncluding vitality, suckle reflex, attitude, fecal score, hydration status, episcleral vessel congestion, ocular/ear abnormalities, nasal discharge, cough, umbilicus condition, sepsis score, and percentage sepsis index – were analyzed using GEE with ordinal logistic regression and a cumulative logit link. Model fit was assessed using the Quasi-likelihood under the Independence Model Criterion (QIC). For each model, the treatment group (CTRL vs HS) was included as a fixed effect. Time (T0–T6) was included as a categorical predictor, with T6 serving as the reference level to evaluate clinical improvement over follow-up. Results were reported as incidence rate ratios (IRRs) or odds ratios (ORs) with 95% confidence intervals (CIs). Statistical significance was set at *p* < 0.05 for all analyses.

## Results

During the study period, from 19 February 2023 to 25 June 2024, 84 diarrheic calves were admitted to the Clinic for Ruminants and Swine. Twenty-four animals did not meet the inclusion criteria and were not enrolled in the study.

### Data at admission

At the time of admission, all enrolled calves exhibited similar age, STP, and blood gas variables, showing no significant differences between the CTRL and HS groups for any of the variables examined (Table 2). Similarly, etiopathogenetic evaluations of fecal samples collected at admission revealed comparable findings between the two groups. Five calves were not subjected to fecal etiological evaluation due to laboratory unavailability. All calves tested positive for *Escherichia coli* (28/28 CTRL; 27/27 HS; *p* value 1.00). *Cryptosporidium* spp. was detected in 16 out of 28 (57.1%) CTRL calves and 10 out of 27 (37.0%) HS calves, with no significant differences observed between the groups (*p* = 0.135). Rotavirus was found in 6 out of 28 (21.4%) CTRL calves and 7 out of 27 (25.9%) HS calves (*p* = 0.695). The positivity rate for coronavirus was also comparable, with CTRL showing 4 of 28 (14.3%) and HS showing 5 of 27 (18.5%; *p* = 0.671). Enterotoxigenic/Shiga toxin-producing *Escherichia coli* (ETEC/STEC) was detected in 3 of 28 (10.7%) CTRL calves but in none of the HS calves; however, this difference did not reach statistical significance (*p* = 0.080). The detection of *Proteus* spp. was similar between the groups, with CTRL at 3 out of 28 (10.7%) and HS at 3 out of 27 (11.1%; *p* = 0.962). Additionally, the antimicrobial susceptibility patterns of *Escherichia coli* did not differ significantly between CTRL and HS calves for any of the tested antimicrobials; detailed percentages and *p*-values are shown in Supplementary Table 2.

**Table 2.** Age, serum total protein (STP), and blood gas variables in 30 CTRL (control) and 30 HS (hazelnut skin-supplemented) calves at admission in a veterinary teaching hospital affected by neonatal calf diarrhea. Normally distributed variables are presented as mean ± standard deviation and were compared using the independent-samples *t*-test; non-normally distributed variables are presented as median with 25th–75th percentiles (P25–P75) and were compared using the Mann–Whitney U test.

Variable	CTRL	HS	*p*-value
*Normally distributed variables*			
Age at admission (days)	8.50 ± 3.24	9.93 ± 3.87	0.125
STP (g/dL)	6.565 ± 1.401	6.754 ± 1.207	0.606
*Non-normally distributed variables*			
Rectal temperature (°C)	38.8 (38.1–39.8)	39.5 (38.7–40.0)	0.216
pH	7.150 (7.045–7.180)	7.140 (7.062–7.233)	0.826
PCO₂ (mmHg)	34.4 (29.1–43.1)	31.2 (26.1–41.4)	0.133
PO₂ (mmHg)	32.8 (25.7–42.2)	37.4 (29.3–43.5)	0.225
cHCO₃ (mmol/L)	12.9 (9.0–17.2)	10.2 (7.7–14.9)	0.192
BE (mmol/L)	–16.0 (–21.1 to –11.0)	–18.2 (–22.4 to –12.3)	0.336
cSO₂ (%)	42.1 (30.1–58.4)	49.0 (31.4–58.0)	0.423
Na⁺ (mmol/L)	128 (123.25–134)	130 (126–133.25)	0.439
K⁺ (mmol/L)	5.18 (4.31–6.90)	5.12 (4.50–6.54)	0.964
Ca²⁺ (mmol/L)	1.33 (1.22–1.42)	1.34 (1.26–1.46)	0.529
Cl⁻ (mmol/L)	102 (96.65–109.5)	104.3 (94.2–109.7)	0.868
cTCO₂ (mmol/L)	12.0 (8.2–15.95)	10.3 (7.95–14.15)	0.375
AGapK (mmol/L)	20.0 (16–21.8)	20.0 (18–23.85)	0.489
Hct (%)	34.0 (27.5–38.5)	30.5 (26.75–37.75)	0.600
STP (g/dL, refractometer)	10.75 (8.85–12.58)	10.00 (9.03–11.88)	0.768
Glucose (mmol/L)	5.33 (4.40–6.50)	5.40 (4.79–6.29)	0.934
Lactate (mmol/L)	1.78 (0.90–5.37)	1.14 (0.64–2.41)	0.073
BUN (mg/dL)	28 (18–53)	30 (23.5–50.5)	0.773
Urea (mmol/L)	13.0 (7.1–20.6)	9.95 (8.6–17.55)	0.475
Creatinine (mg/dL)	1.93 (1.19–3.52)	1.85 (1.49–3.15)	0.775

PCO₂: partial pressure of carbon dioxide; PO₂: partial pressure of oxygen; cHCO₃: bicarbonate concentration; BE: base excess; cSO₂: oxygen saturation; Na⁺: sodium; K⁺: potassium; Ca²⁺: ionized calcium; Cl⁻: chloride; cTCO₂: total carbon dioxide; AGapK: anion gap corrected for potassium; Hct: hematocrit; BUN: blood urea nitrogen.

These results highlight the homogeneity of both study groups at enrollment, which facilitated a more objective assessment of HS supplementation outcomes.

### Clinical status during hospitalization

During hospitalization, clinical status evaluations did not show a difference between groups. The descriptive statistics for repeated measures were reported in Table 3. In our Clinic's assessment of clinical categorization for calves affected by NCD, we analyzed various indicators, including demeanor score, suckle reflex, fecal score, and hydration status (Table 1). The statistical model demonstrated no significant effect of the group on the demeanor score (*p* = 0.806); however, it revealed a temporally substantial impact (*p* < 0.001). Early time points showed that calves exhibited considerably lower odds of being categorized in low vitality status when compared to T6, with OR highlighting this decline: T0 (OR = 0.03, 95% CI 0.02–0.07, *p* < 0.001), T1 (OR = 0.26, 95% CI 0.13–0.54, *p* < 0.001), T2 (OR = 0.34, 95% CI 0.18–0.67, *p* = 0.002), and T3 (OR = 0.48, 95% CI 0.25–0.91, *p* = 0.024). Starting from T4, demeanor scores showed no significant differences when compared with T6 (*p* > 0.05). For the suckle reflex, the group effect was also non-significant (*p* = 0.799), whereas time presented a highly significant relationship (*p* < 0.001). In contrast to T6, the odds of impaired suckle reflex were markedly higher at T0 (OR = 36.00, 95% CI 11.09–116.89, *p* < 0.001), with subsequent time points showing a progressive decrease: T1 (OR = 5.52, 95% CI 1.99–15.26, *p* = 0.001), T2 (OR = 3.73, 95% CI 1.42–9.85, *p* = 0.008), and T3 (OR = 2.83, 95% CI 1.28–6.25, *p* = 0.010). As with the demeanor score, there was no significant difference from T6 onward (*p* > 0.05). Regarding the fecal score, group differences were not detected (*p* = 0.885), but, again, time was strongly associated with improvement (*p* < 0.001). Early time points indicated a higher likelihood of poorer fecal scores compared to T6: T0 (OR = 14.44, 95% CI 6.80–30.68), T1 (OR = 10.61, 95% CI 5.10–22.05), and T2 (OR = 9.71, 95% CI 4.89–19.28), all with *p*-values < 0.001. Even at T3 (OR = 3.89, 95% CI 2.05–7.41, *p* < 0.001), T4 (OR = 1.98, 95% CI 1.17–3.34, *p* = 0.010), and T5 (OR = 2.06, 95% CI 1.42–2.99, *p* < 0.001), calves continued to show increased odds of worse fecal scores, indicating a marked transition toward normalization of fecal consistency over time for both groups. For hydration status, the group effect was again non-significant (*p* = 0.399), whereas time was significant (*p* < 0.001). At T0, calves were significantly more likely to exhibit moderate to severe dehydration compared with T6 (OR = 67.93, 95% CI 24.49–188.42, *p* < 0.001). The odds of experiencing worse hydration remained elevated at T1 (OR = 2.11, 95% CI 1.02–4.33, *p* = 0.043) and T3 (OR = 2.30, 95% CI 1.10–4.83, *p* = 0.027). No significant differences were observed at a later time point compared with T6 (*p* > 0.05). The descriptive statistics for clinical data were reported in Supplementary Table 4.

**Table 3.** Repeated measurements in 30 CTRL (control) calves and 30 HS (supplemented with hazelnut skin) during the study period; from admission (T0) to the sixth day of hospitalization (T6). Variables are presented as median with 25th–75th percentiles (P25–P75).

Time	Weight (kg)	Milk intake (L/day)	Rectal temperature (°C)	Sepsis score
**CTRL**				
T0	35.0 (33.0–39.0)		39.2 (38.5–39.8)	4.8 (3.7–5.2)
T1	38.0 (36.0–41.5)	5.0 (3.45–6.0)	38.8 (38.6–39.05)	2.7 (2.7–4.8)
T2	38.0 (35.5–42.0)	5.5 (3.38–6.0)	38.8 (38.5–39.1)	2.7 (2.7–2.7)
T3	37.0 (35.0–40.0)	5.0 (4.0–6.0)	38.8 (38.6–39.1)	2.7 (2.7–2.7)
T4	37.0 (35.0–40.0)	6.0 (4.0–6.0)	38.7 (38.5–39.0)	2.7 (2.7–2.7)
T5	37.0 (35.0–42.0)	5.75 (4.0–6.0)	38.8 (38.48–39.23)	2.7 (2.7–2.7)
T6	37.5 (35.0–41.25)	6.0 (4.38–6.0)	38.8 (38.58–39.13)	2.7 (2.7–2.7)
**HS**				
T0	36.0 (32.5–39.0)		39.2 (38.6–39.8)	4.8 (2.7–5.2)
T1	38.0 (33.75–40.25)	5.0 (3.25–6.0)	38.7 (38.5–39.0)	2.7 (2.7–4.8)
T2	39.0 (35.0–40.0)	4.7 (4.0–6.0)	38.7 (38.4–39.1)	2.7 (2.7–4.8)
T3	39.0 (35.0–41.0)	5.0 (3.5–6.0)	38.6 (38.4–39.2)	2.7 (2.7–2.7)
T4	39.0 (35.0–42.0)	6.0 (4.0–6.0)	38.7 (38.5–39.0)	2.7 (2.7–2.7)
T5	39.0 (36.0–41.0)	6.0 (4.5–6.0)	38.65 (38.4–38.83)	2.7 (2.7–2.7)
T6	39.0 (36.75–41.0)	6.0 (4.38–6.0)	38.8 (38.33–39.03)	2.7 (1.2–2.7)

**Table 4.** Serum antioxidant capacity using the oxygen-adsorbent test (OXY) and reactive oxygen metabolites (d-ROMs) values from admission (T0) to the sixth day of hospitalization (T6) in 30 CTRL (control) calves and 30 HS (supplemented with hazelnut skin). OXY values are expressed in mean ± standard deviation (µmol HClO/mL); d-ROMs as median with 25th–75th percentiles (H₂O₂/dL).

Oxidation status marker	CTRL	HS
**OXY**		
T0	345.7 ± 77.3	369.0 ± 89.1
T6	385.1 ± 86.3	371.5 ± 100.2
**d-ROMS**		
T0	24.23 (15.75–106.65)	24.75 (16.37–106.48)
T6	21.56 (13.20–135.90)	26.74 (17.01–123.97)

### Weight gain, milk intake during hospitalization, number of fluid treatments administered, mortality rate, and hospitalization time

Weight gain from admission until day six of hospitalization was comparable between groups (CTRL: 2.04 ± 2.27 kg; HS: 2.08 ± 2.93 kg; *p* = 0.958). The GEE model showed no group effect (*p* = 0.814) but an apparent time effect (*p* < 0.001) in both groups. At earlier time points, calves had significantly lower body weight compared with T6, consistent with growth during follow-up (T0 vs T6: IRR = 0.93, 95% CI 0.91–0.95, *p* < 0.001).

No significant differences were observed between groups for hospitalization time (CTRL: median 8 days, P25–P75: 7–11; HS: median 9 days, P25–P75: 8–12; *p* = 0.294) and for the number of fluid treatments administered (CTRL: median 1, P25–P75: 1–2; HS: median 1.5, P25–P75: 1–2; *p* = 0.334). Mortality at day 6 was identical in CTRL and HS calves: 26/30 (86.7%) survived, and 4/30 (13.3%) died in both groups (*p* = 1.000). In the CTRL group, 23/30 calves (76.7%) were discharged, and 7/30 (23.3%) died during the addition time, compared with 24/30 (80%) discharged calves and 6/30 (20%) died calves in the HS group (*p* = 0.754).

For milk intake, there was no group effect (*p* = 0.979), whereas time was significantly associated with intake (*p* < 0.001). Intake at T1–T3 and T5 was lower than at T6 (IRR = 0.87, 95% CI 0.81–0.94, *p* < 0.001).

### Sepsis score

For the sepsis score, the GEE model showed no group effect (*p* = 0.627), while time had a marked influence (*p* < 0.001). Compared with T6, calves at T0 had substantially higher odds of belonging to higher sepsis score categories (IRR = 32.52, 95% CI 13.84–76.42, *p* < 0.001), with still increased odds at T1 (IRR = 5.93, 95% CI 2.68–13.12, *p* < 0.001), T2 (IRR = 3.08, 95% CI 1.38–6.89, *p* = 0.006) and T3 (IRR = 2.69, 95% CI 1.28–5.65, *p* = 0.009). At T4 and T5, sepsis score categories did not differ significantly from T6 (*p* > 0.050). Similarly, for the percentage sepsis index, no group effect was observed (*p* = 0.976), whereas time was strongly associated with the distribution of sepsis index (*p* < 0.001). Compared with T6, calves at T0 had markedly higher odds of belonging to higher sepsis index (OR = 21.91, 95% CI 10.62–45.19, *p* < 0.001), with elevated odds still present at T1 (OR = 3.72, 95% CI 1.99–6.96, *p* < 0.001), T2 (OR = 2.26, 95% CI 1.21–4.25, *p* = 0.011) and T3 (OR = 2.12, 95% CI 1.15–3.91, *p* = 0.016). No significant differences were observed at T4–T5 (all *p* > 0.05). No statistically significant differences were observed between groups or over time in the data used to evaluate the sepsis score.

### Antioxidant status

At baseline (T0), OXY and d-ROMs values were comparable between groups. Similarly, at T6, no significant differences were observed, and no substantial changes could be attributed to HS treatment (see Table 4). Generalized estimating equation models further corroborated the lack of a treatment effect on both antioxidant and pro-oxidant biomarkers. Regarding OXY, the group effect approached statistical significance (*p* = 0.050), suggesting only a marginal difference between control (CTRL) and HS calves over time (CTRL vs HR; IRR −28.69; 95% CI: −57.37 to −0.009). For d-ROMs, neither the group effect nor the time effect reached statistical significance (*p* = 0.696 and *p* = 0.064, respectively).

## Discussion

This study aimed to evaluate the efficacy of HS extract administration as an adjunct to conventional therapy in hospitalized dairy heifers diagnosed with NCD. The research specifically aimed to elucidate whether this intervention could significantly improve clinical health outcomes and accelerate recovery trajectories. The rationale behind this study was based on information regarding the impact of polyphenolic compounds on intestinal health. The hypothesized protective mechanisms of polyphenols in mitigating intestinal pathologies are primarily due to their phenolic hydroxyl groups. These groups are believed to mediate a spectrum of biochemical activities pertinent to intestinal health, such as promoting the proliferation of beneficial microbiota that are integral to maintaining intestinal barrier integrity (Makarewicz et al. 2021), as well as exerting pronounced anti-inflammatory effects within the gastrointestinal tract (Gessner et al. 2012; Ismail et al. 2025). Although this, our study revealed no statistically significant differences between treatment groups regarding either clinical or hematological parameters. Most clinical data improved between 3 and 5 days after admission, similarly across the two groups, indicating that the established therapeutic protocol had an impact on the enrolled animals, without any additional benefit from HS administration. In our clinic, the standard treatment protocol for hospitalized calves with NCD includes fluid therapy combined with antimicrobial administration, and the rationale for this choice is based on several considerations: recent literature suggests that antibiotics should be reserved for specific cases of NCD under field conditions (Gomez et al. 2021); however, hospitalized calves face a considerably higher risk of bacteremia and septicemia (Pas et al. 2023). Therefore, adopting a systematic approach to antimicrobial administration for hospitalized calves with NCD may be advisable, particularly given the presence of animals from various herds, which heightens their exposure to significant epidemiological pressures and which increases their exposure to significant epidemiological pressures, as well as the frequent manifestation of systemic clinical signs among these patients, thereby substantiating the judicious use of antimicrobials (Smith 2015). Additionally, a clinical trial must ensure that the subject groups are as homogeneous as possible. In studies comparing clinical signs between treated and untreated groups, administering antimicrobials to only a subset of individuals could unintentionally affect fecal scores (Berge et al. 2009) and other NCD-related clinical indicators, as well as overall mortality and morbidity rates between the groups. To minimize this potential bias, we administered 150 mg/kg of long-acting amoxicillin (Bymoxyl LA®, TREI S.p.A., a LIVISTO company, Modena, Italy) intramuscularly to all calves in both groups upon admission, with a second dose given 48 h later.

These findings diverge from those of the two existing studies that assessed tannic compounds within a therapeutic rather than a prophylactic framework for NCD. These studies reported that phytotherapeutic compounds may reduce the duration of diarrhea and improve weight gain in affected calves (Bonelli et al. 2018; Wang et al. 2022). Structural distinctions in the present study design could have contributed to the divergent results. Notably, Wang et al. (2022) employed a larger sample size but adopted substantially different methodologies and eligibility criteria. A marked contrast in outcomes between antibiotic and phytotherapeutic compound treatments characterized their investigation. The observed reductions in fecal scores in calves treated with the tannic complex in that study may have been confounded by concurrent antimicrobial use in the other study group, which is known to affect fecal consistency (Berge et al. 2009), and that, moreover, prompted us to include the antibiotic in both of our study groups. Moreover, while Wang et al. (2022) enrolled field cases characterized by mild clinical presentations, the present study cohort comprised predominantly severely ill, hospitalized calves – a factor that may inherently diminish the observable impact of adjunctive phytotherapeutic strategies. In a similar vein, Bonelli et al. (2018) restricted their analysis to non-severe cases, thereby obviating the need for ancillary pharmacological or intravenous-based therapies during the study period. By contrast, the current trial excluded calves amenable to oral rehydration and enrolled exclusively those requiring intravenous fluid administration. Accordingly, this investigation is notable as the first to systematically examine polyphenol supplementation in a cohort of calves with NCD requiring medical intervention (see the severe median values for acid-base disarrangements in both groups in Table 2). These conflicting findings also underscore a fundamental limitation inherent to this category of scientific investigation, both in evaluating the therapeutic efficacy of phytotherapeutic compounds and in assessing their prophylactic utility for mitigating the incidence of NCD. A central methodological concern is the operational definition of the disease: diagnostic criteria often limit themselves to fecal scoring alone, whereas a standardized evaluation of calf behavior and hydration status may be required to achieve a more precise diagnosis of NCD (Wilson et al. 2023).

Another principal methodological challenge lies in the rational determination of both the dosage and the temporal framework for administering these compounds. To address this, we conducted an extensive review of the relevant literature to inform the selection of our dosing regimen. The 10 g/day dosage over 5 days was extrapolated from previous research using polyphenolic extracts derived from diverse botanical sources. For instance, Bonelli et al. (2018) administered 10 g of chestnut extract, characterized by a tannin content of 224 mg/g and a GAE value of 56.99 g/100 g in NCD-affected calves. Turini et al. (2022) assessed the effects of a phytogenic additive containing 58 mg/g polyphenols, administered daily at 30 g for 24 days of life in calves, yielding a cumulative daily intake of 1710 mg polyphenols, yet failed to confer a preventive benefit against the onset of neonatal diarrhea. Relevant evidence from other species further contextualizes the dosing rationale. Wang et al. (2019) supplemented pregnant and lactating sows with grape seed extract at dosages of 200 and 300 mg/kg body weight, resulting in improved piglet survival rates. Qualitative analysis of the HS extract employed in the present study revealed a polyphenol concentration of 850 mg/g, culminating in a total polyphenol dose of 8500 mg per calf (approximately 20 mg/kg), which is analogous to the effective dose reported in the porcine model and exceeds those used in previous bovine studies focused on prophylaxis. The dosing strategy adopted herein is predicated on the hypothesis that polyphenol supplementation may exert therapeutic effects in established disease rather than merely serve a preventive function.

Notwithstanding careful consideration of dosing parameters, HS administration did not yield a measurable impact on clinical outcomes in our study cohort. In addition to the pronounced severity of underlying conditions in the enrolled population, a plausible explanation for the absence of observable therapeutic benefit following HS administration may lie in the comparatively brief treatment duration. The five-day administration interval was selected primarily because it aligns with the conventional timeframe for clinical recovery – defined by amelioration of demeanor, restoration of autonomous standing, and improvement of the sucking reflex – after intervention in the most severe diarrheic cases (Boccardo et al. 2019; Welk et al. 2025). Moreover, this period was determined to balance cost-efficiency and logistical feasibility, thereby supporting the practical application of these therapeutic approaches in realistic clinical settings. Conversely, previous investigations involving healthy calves that evaluated the prophylactic potential of polyphenolic compounds reported beneficial alterations in clinical and antioxidant parameters only when supplementation was maintained for 15 to 70 days (Oliveira et al. 2010; Boccardo et al. 2026). Within the context of NCD therapy, it is impractical to maintain such prolonged supplementation regimens. Nonetheless, extant literature predominantly indicates that polyphenol supplementation used in a prophylactic manner exerts only a marginal effect in mitigating the clinical manifestations of NCD (Kodithuwakku et al. 2021; Turini et al. 2022; McNeil et al. 2023; Boccardo et al. 2026), thereby casting doubt on the therapeutic utility of these agents.

This investigation is subject to several methodological limitations. Although the original study design aimed to implement a blinded assessment of both experimental groups, the administration of HS resulted in a distinct reddish discoloration of the feces, thereby precluding adequate blinding and potentially introducing observer bias. Furthermore, the study deliberately selected clinically ill calves for inclusion, and HS extract was incorporated as an adjunctive agent within a comprehensive therapeutic protocol, with HS administration limited to 5 days. These factors collectively raise the possibility that extended HS supplementation and/or its application in less severely affected subjects may have yielded more pronounced clinical benefits.

In conclusion, our study provides evidence supporting the hypothesis that HS supplementation has no beneficial effects when added to standard infusion therapy in calves with NCD.

## Supplementary Material

Supplementary Material Supplementary materials.docx

## Data Availability

The datasets generated and analyzed during the current study are available from the corresponding author upon reasonable request.
