# Supplementary material for: Effects on health and antioxidant status of oral supplementation of hazelnut skin extract in dairy calves with neonatal diarrhea: a randomized clinical trial in hospitalized patients
Source: Vet Q. 2026 Oct 14;46(1):2691428. doi: 10.1080/01652176.2026.2691428 (PMC13670267; doi:10.1080/01652176.2026.2691428)
Supplement: Supplementary file 1 [file TVEQ_A_2691428_SM4214.docx]

**Supplementary materials**

*Supplementary Table 1.* Description of clinical scoring method used to inform the scoring sheet for sepsis score and prediction of bacteremia

| Score | Clinical signs |
| --- | --- |
| Fecal score |  |
| 0 | Normal feces |
| 1 | Softer than normal but no diarrhea on tail |
| 2 | Diarrhea, but no profuse; wet tail |
| 3 | Profuse watery diarrhea or any indication of blood or fibrin in feces |
| Hydration status |  |
| 0 | Normal hydration, skin tent <2 s |
| 1 | Moderate dehydration, eyeball slightly sunken, and skin tent >2 s but <4 s |
| 2 | Obvious dehydration, eyes sunken, dry nose, skin tent >5 s |
| 3 | Severe dehydration, eyes very sunken with a easily perceptible distance between the eyeball and the eyelid, skin tent persists |
| Episcleral vessels |  |
| 0 | Normal (<2) they do not reach the limbus |
| 1 | Greater in number (<4), at least 1 reaches the limbus, color is still pink, size is normal |
| 2 | Greater in number (>4), at least 2 reach the limbus, color is red, size is mildly increased |
| 3 | Greater in number (>6), at least 3 reach the limbus, color is purple, size is greatly increased |
| Umbilicus |  |
| 0 | Normal, pencil size, dry and painless |
| 1 | Bigger than normal but dry and painless |
| 2 | Bigger than normal, wet or painful |
| 3 | Bigger than normal, with pus draining and evidence of pain |
| Attitude |  |
| 0 | Normal behavior, alert, gets up when approached, interested in surroundings |
| 1 | Depressed, must be stimulated to get up |
| 2 | Gets up only with help |
| 3 | Unable to stand, even with help |

*Fecteau, G., Pare, J., Van Metre, D. C., Smith, B. P., Holmberg, C. A., Guterbock, W., & Jang, S. (1997). Use of a clinical sepsis score for predicting bacteremia in neonatal dairy calves on a calf rearing farm. The Canadian Veterinary Journal, 38(2), 101.

*Supplementary Table 2.* **Antimicrobial susceptibility profiles in 28 control (CTRL) and 27 hazelnut skin-supplemented (HS) calves at admission in a veterinary teaching hospital affected by neonatal calf diarrhea. Antimicrobial susceptibility results are expressed as frequency (percentage) for each interpretive category (susceptible, intermediate, resistant). Antimicrobials with no variability (all isolates classified in the same category) are reported as n/a because statistical comparison was not applicable.**

|  | CTRL | | | HS | | |  |
| --- | --- | --- | --- | --- | --- | --- | --- |
| Antimicrobial | Susceptible | Intermediate | Resistant | Susceptible | Intermediate | Resistant | p-value |
| **Aminopenicillins** | 24 (88.9%) | 0 | 3 (11.1%) | 21 (77.8%) | 0 | 6 (22.2%) | 0.273 |
| **Amoxicillin–clavulanate** | 7 (25.0%) | 4 (14.3%) | 17 (60.7%) | 10 (37.0%) | 3 (11.1%) | 14 (51.9%) | 0.624 |
| **Ampicillin** | 28 (100%) | 0 | 0 | 27 (100%) | 0 | 0 | n/a |
| **Cefazolin** | 9 (33.3%) | 14 (51.9%) | 4 (14.8%) | 8 (29.6%) | 13 (48.1%) | 6 (22.2%) | 0.780 |
| **Cefotaxime** | 4 (100%) | 0 | 0 | 3 (100%) | 0 | 0 | n/a |
| **Colistin** | 4 (14.8%) | 0 | 23 (85.2%) | 1 (3.7%) | 0 | 26 (96.3%) | 0.159 |
| **Enrofloxacin** | 16 (57.1%) | 10 (35.7%) | 2 (7.1%) | 13 (48.1%) | 11 (40.7%) | 3 (11.1%) | 0.763 |
| **Florfenicol** | 17 (60.7%) | 9 (32.1%) | 2 (7.1%) | 16 (59.3%) | 9 (33.3%) | 2 (7.4%) | 0.994 |
| **Flumequine** | 21 (77.8%) | 4 (14.8%) | 2 (7.4%) | 18 (66.7%) | 7 (25.9%) | 2 (7.4%) | 0.592 |
| **Gentamicin** | 16 (59.3%) | 0 | 11 (40.7%) | 10 (37.0%) | 2 (7.4%) | 15 (55.6%) | 0.135 |
| **Kanamycin** | 24 (88.9%) | 0 | 3 (11.1%) | 21 (77.8%) | 0 | 6 (22.2%) | 0.273 |
| **Sulfisoxazole** | 25 (100%) | 0 | 0 | 24 (100%) | 0 | 0 | n/a |
| **Tetracycline** | 25 (92.6%) | 0 | 2 (7.4%) | 22 (81.5%) | 0 | 5 (18.5%) | 0.224 |
| **Trimethoprim–sulfonamide** | 25 (89.3%) | 0 | 3 (10.7%) | 21 (77.8%) | 0 | 6 (22.2%) | 0.249 |
| **Ceftiofur** | 1 (100%) | 0 | 0 | – | – | – | n/a |
| **Penicillin** | 1 (100%) | 0 | 0 | – | – | – | n/a |

*Supplementary Tables 3.*

Demeaner score* in 30 CTRL (control) calves and 30 HS (supplemented with hazelnut husks) during the study period; from admission (T0) to the sixth day of hospitalization (T6). The variables are presented as frequency (percentage).

| Time | Score 1 | Score 2 | Score 3 | Score 4 | Score 5 |
| --- | --- | --- | --- | --- | --- |
| CTRL | | | | | |
| T0 | 1 (9.7) | 10 (32.3) | 11 (35.5) | 4 (12.9) | 3 (9.7) |
| T1 | 0 (0.0) | 3 (10.3) | 6 (20.7) | 10 (34.5) | 10 (34.5) |
| T2 | 0 (0.0) | 1 (3.4) | 3 (10.3) | 14 (48.3) | 11 (37.9) |
| T3 | 0 (0.0) | 1 (3.7) | 3 (11.1) | 9 (33.3) | 14 (51.9) |
| T4 | 0 (0.0) | 0 (0.0) | 3 (11.1) | 5 (18.5) | 19 (70.4) |
| T5 | 0 (0.0) | 0 (0.0) | 2 (7.7) | 7 (26.9) | 17 (65.4) |
| T6 | 0 (0.0) | 0 (0.0) | 1 (3.8) | 8 (30.8) | 17 (65.4) |
| HS | | | | | |
| T0 | 2 (6.9) | 10 (34.5) | 7 (24.1) | 7 (24.1) | 3 (10.3) |
| T1 | 1 (3.3) | 0 (0.0) | 8 (26.7) | 9 (30.0) | 12 (40.0) |
| T2 | 0 (0.0) | 1 (3.7) | 5 (18.5) | 11 (40.7) | 10 (37.0) |
| T3 | 0 (0.0) | 2 (7.4) | 4 (14.8) | 8 (29.6) | 13 (48.1) |
| T4 | 0 (0.0) | 3 (11.1) | 4 (14.8) | 10 (37.0) | 14 (51.9) |
| T5 | 0 (0.0) | 0 (0.0) | 10 (38.5) | 16 (61.5) | 0 (0.0) |
| T6 | 0 (0.0) | 1 (3.8) | 1 (3.8) | 7 (26.9) | 17 (65.4) |

*Score 5, standing securely without assistance, curious, alert; Score 4, standing up after encouragement, weak, ‘sad calf’; Score 3, sternal recumbency, standing after lifting, ‘drunken gait’, insecure posture; Score 2 permanent sternal/costal recumbency; Score 1 lateral recumbency, comatose.

Suckling reflex score* in 30 CTRL (control) calves and 30 HS (supplemented with hazelnut husks) during the study period; from admission (T0) to the sixth day of hospitalization (T6). The variables are presented as frequency (percentage).

| Time | Score 2 | Score 1 | Score 0 |
| --- | --- | --- | --- |
| CTRL | | | |
| T0 | 18 (58.1) | 9 (29.0) | 4 (12.9) |
| T1 | 10 (34.5) | 15 (51.7) | 4 (13.8) |
| T2 | 8 (27.6) | 15 (51.7) | 6 (20.7) |
| T3 | 7 (25.9) | 13 (48.1) | 7 (25.9) |
| T4 | 10 (37.0) | 12 (44.4) | 5 (18.5) |
| T5 | 7 (26.9) | 12 (46.2) | 7 (26.9) |
| T6 | 8 (30.8) | 14 (53.8) | 4 (15.4) |
| HS | | | |
| T0 | 18 (62.1) | 9 (31.0) | 2 (6.9) |
| T1 | 14 (46.7) | 12 (40.0) | 4 (13.3) |
| T2 | 11 (40.7) | 12 (44.4) | 4 (14.8) |
| T3 | 9 (33.3) | 11 (40.7) | 7 (25.9) |
| T4 | 10 (37.0) | 13 (48.1) | 4 (14.8) |
| T5 | 10 (38.5) | 8 (30.8) | 8 (30.8) |
| T6 | 12 (46.2) | 11 (42.3) | 3 (11.5) |

*Score 2, absent or chewing movements; Score 1, weak; Score 0, strong.

Hydration score in 30 CTRL (control) calves and 30 HS (supplemented with hazelnut husks) during the study period; from admission (T0) to the sixth day of hospitalization (T6). The variables are presented as frequency (percentage).

| Time | Score 0 | Score 1 | Score 2 | Score 3 |
| --- | --- | --- | --- | --- |
| CTRL | | | | |
| T0 | 2 (6.5) | 9 (29.0) | 12 (38.7) | 8 (25.8) |
| T1 | 2 (6.9) | 10 (34.5) | 11 (37.9) | 6 (20.7) |
| T2 | 3 (10.3) | 9 (31.0) | 9 (31.0) | 8 (27.6) |
| T3 | 5 (18.5) | 11 (40.7) | 8 (29.6) | 3 (11.1) |
| T4 | 6 (22.2) | 12 (44.4) | 8 (29.6) | 1 (3.7) |
| T5 | 10 (38.5) | 12 (46.2) | 3 (11.5) | 1 (3.8) |
| T6 | 11 (42.3) | 11 (42.3) | 3 (11.5) | 1 (3.8) |
| HS | | | | |
| T0 | 3 (10.3) | 8 (27.6) | 10 (34.5) | 8 (27.6) |
| T1 | 4 (13.3) | 11 (36.7) | 10 (33.3) | 5 (16.7) |
| T2 | 4 (14.8) | 10 (37.0) | 8 (29.6) | 5 (18.5) |
| T3 | 7 (25.9) | 11 (40.7) | 7 (25.9) | 2 (7.4) |
| T4 | 8 (29.6) | 11 (40.7) | 7 (25.9) | 1 (3.7) |
| T5 | 10 (38.5) | 9 (34.6) | 6 (23.1) | 1 (3.8) |
| T6 | 12 (46.2) | 10 (38.5) | 3 (11.5) | 1 (3.8) |

*Score 0, normal hydration, upper eyelid skin tent; Score 1, moderate dehydration, eyeball slightly sunken (1–2 mm), or upper eyelid skin tent >2 s; Score 2, obvious dehydration, sunken eyes (3–4 mm), upper eyelid skin tent >5 s (estimated loss of body mass 6–8%); Score 3 Severe sunken eyes with an easily perceptible distance between the eyeball and the eyelid (≥5 mm), cold legs and oral cavity, dry mouth and nose, upper eyelid skin tent persists (estimated loss of body mass ≥9%)

Fecal score (from Rebaud et al., 2020)* in 30 CTRL (control) calves and 30 HS (supplemented with hazelnut husks) during the study period; from admission (T0) to the sixth day of hospitalization (T6). The variables are presented as frequency (percentage).

| Time | Score 0 | Score 1 | Score 2 | Score 3 |
| --- | --- | --- | --- | --- |
| CTRL | | | | |
| T0 | 1 (3,2%) | 1 (3,2%) | 13 (41,9%) | 16 (51,6%) |
| T1 | 1 (3,4%) | 1 (3,4%) | 11 (37,9%) | 16 (55,2%) |
| T2 | 0 (0,0%) | 2 (6,9%) | 18 (62,1%) | 9 (31,0%) |
| T3 | 1 (3,7%) | 6 (22,2%) | 15 (55,6%) | 5 (18,5%) |
| T4 | 2 (7,4%) | 11 (40,7%) | 12 (44,4%) | 2 (7,4%) |
| T5 | 2 (7,7%) | 10 (38,5%) | 11 (42,3%) | 3 (11,5%) |
| T6 | 5 (19,2%) | 12 (46,2%) | 6 (23,1%) | 3 (11,5%) |
| HS | | | | |
| T0 | 0 (0,0%) | 2 (6,9%) | 11 (37,9%) | 16 (55,2%) |
| T1 | 0 (0,0%) | 6 (20,0%) | 12 (40,0%) | 12 (40,0%) |
| T2 | 1 (3,7%) | 3 (11,1%) | 8 (29,6%) | 15 (55,6%) |
| T3 | 3 (11,1%) | 4 (14,8%) | 13 (48,1%) | 7 (25,9%) |
| T4 | 6 (22,2%) | 4 (14,8%) | 12 (44,4%) | 5 (18,5%) |
| T5 | 7 (26,9%) | 5 (19,2%) | 6 (23,1%) | 8 (30,8%) |
| T6 | 9 (34,6%) | 6 (23,1%) | 8 (30,8%) | 3 (11,5%) |

* Renaud, D. L., Buss, L., Wilms, J. N., & Steele, M. A. (2020). Is fecal consistency scoring an accurate measure of fecal dry matter in dairy calves?. Journal of Dairy Science, 103(11), 10709-10714.
